# Editorial: Molecular interactions between bacterial pathogens and plants: selected contributions to the 14th International Conference on Plant Pathogenic Bacteria (14th ICPPB)

**DOI:** 10.3389/fpls.2023.1345785

**Published:** 2023-12-11

**Authors:** Roberto Buonaurio, Chiaraluce Moretti, Vittoria Catara, Michelle T. Hulin, Guido Sessa, George W. Sundin

**Affiliations:** ^1^ Dipartimento di Scienze Agrarie, Alimentari e Ambientali, Università degli Studi di Perugia, Perugia, Italy; ^2^ Department of Agriculture, Food and Environment, University of Catania, Catania, Italy; ^3^ The Sainsbury Laboratory, Norwich Research Park, Norwich, United Kingdom; ^4^ School of Plant Sciences and Food Security, Tel-Aviv University, Tel-Aviv, Israel; ^5^ Department of Plant Soil & Microbial Sci, Michigan State University, E Lansing, MI, United States

**Keywords:** bacterial diagnosis, bacterial genomes, bacterial taxonomy, effectors, phytopathogenic bacteria

‘The Impact of Plant Pathogenic Bacteria on Global Plant Health’ was the slogan accompanying the 14^th^ International Conference on Plant Pathogenic Bacteria (ICPPB), held from 3^rd^ to 8^th^ July, 2022 at Assisi (Italy), which attracted 196 delegates from 37 countries across most continents. Among the 9 scientific sessions of the conference program, the session: Molecular interaction Between Bacterial Pathogens and Plants has been proposed as argument for the present Research Topic, a subject that attracted the attention of many plant pathologists. This Research Topic is dedicated to Nicola Sante Iacobellis co-Chair of the 14^th^ ICPPB and Guido Sessa co-Editor of the present Research Topic who prematurely passed away (**Figure 1**).

**Figure 1:**
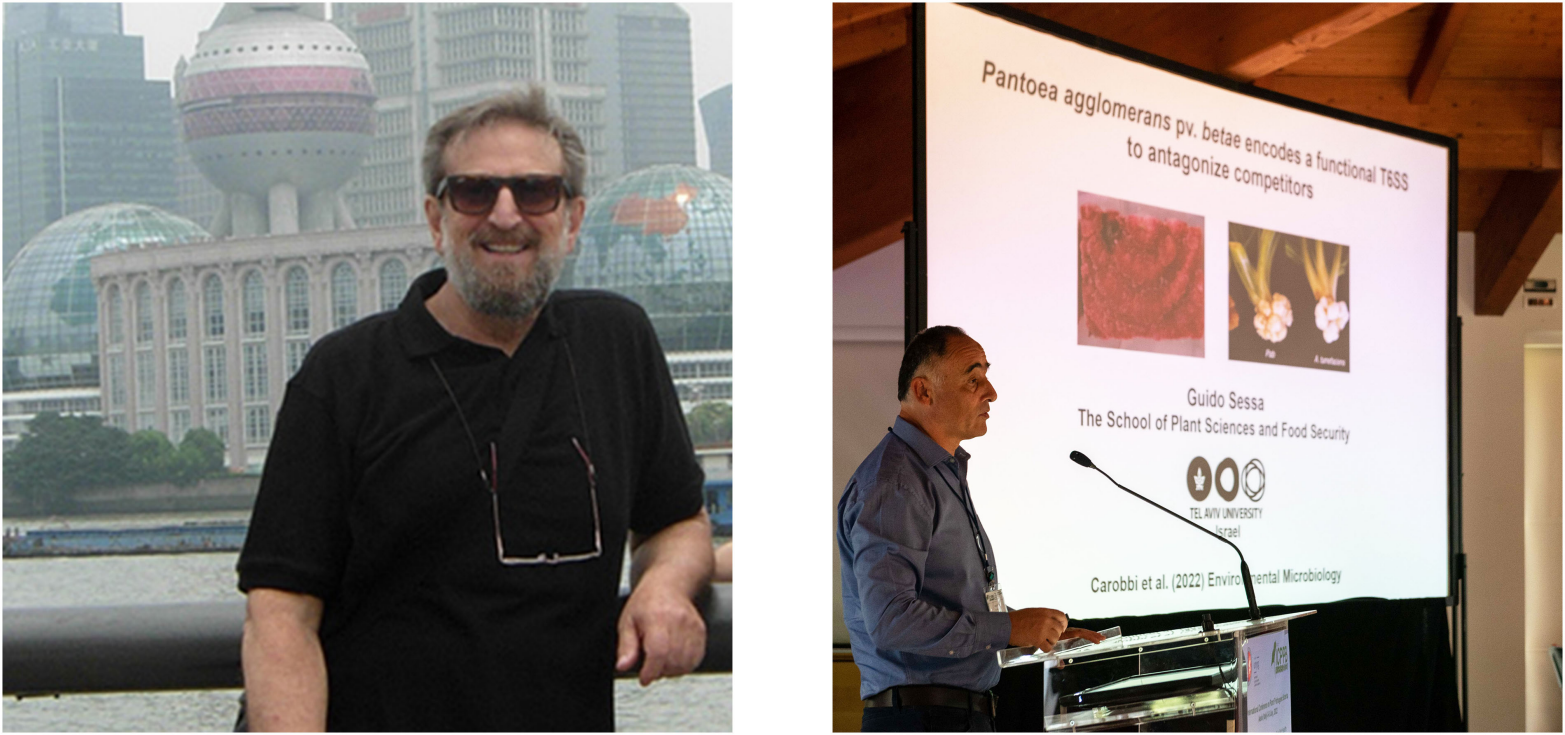
Nicola Sante Iacobellis (1949-2022) at the 13^th^ ICPPB-Shanghai-China (2014). Guido Sessa (1964-2023) at the 14^th^ ICPPB-Assisi-Italy (2022). Written informed consent was obtained from the individual(s) for the publication of any identifiable images or data included in this article.

What factors determine and modulate the pathogenicity and virulence of phytopathogenic bacteria in plants is a main question that phytobacteriologists have asked themselves whilst studying plant-bacterium interactions. The tremendous increase in the number of whole-genome sequences of bacterial strains and the powerful tools now available for interpreting sequencing data are providing essential information to answer to this question. The attention is mainly focused on type-3 effectors (T3Es) which in the form of a cocktail are injected into plant cells by many phytopathogenic bacteria through a type-III secretion system and are able to manipulate host processes for the benefit of the bacteria and thus promote disease development.

Using Effectidor: an automated machine-learning-based web server for the prediction of T3Es (Wagner et al., 2022), Wagner et al. discover 6 new T3Es in the newly sequenced genome of *Xanthomonas hortorum* pv. pelargonii isolated in Israel and also in *Xanthomonas fragariae*. They also experimentally validated the translocation of these new T3Es, which are added to the list of many bacterial effectors known today.

Guido Sessa’s group fully assembled the genomes of tumorigenic bacteria: *Pantoea agglomerans* pv. betae (Pab) which provokes disease on gypsophila and sugar beet and *P. agglomerans* pv. gypsophilae (Pag) on gypsophila (Manulis and Barash, 2003), and carried out a comparative sequence analysis of the Pab and Pag pathogenicity plasmids (Geraffi et al.). They found two novel type-3 chaperones of the ShcV and CesT families present in both pathovars with high similarity. Geraffi et al. also identified insertion sequences and transposons that may have contributed to the evolution of the two pathovars.

Orfei et al. sequenced the complete genome of *Pseudomonas syringae* pv. tomato strain DAPP-PG 215, race 0 (Buonaurio et al., 1996) and demonstrated that this strain has a race 1 genotype but displays a race 0 phenotype. The DAPP-PG 215 strain is phylogenetically closely related to a number of race 1 strains; its genome bearing *hopW1* and *avrA* genes, which are considered as diagnostic markers for race 1 (Jones et al., 2015), and not the *hopN1* gene, a marker for race 0 strains. However, the genome harbors a complete ortholog of *avrPto1*, which allows the strain to display a race 0 phenotype and is within a prophage: a mobile genetic element that can be responsible for horizontal gene transfer.

Studies aimed at the host plant in the plant-bacteria interactions are mainly focused on plant defense responses and try to answer how the bacterium evade the plant defenses to cause disease. These studies open up opportunities to identify new strategies to fight plant bacterial diseases.

*Walls Are Thin1* (*WAT1*) is a susceptible plant gene encoding a tonoplast localized vacuolar auxin transporter first found in Arabidopsis that enables the infection process of vascular pathogens; the Arabidopsis *wat1-1* mutant was found to be resistant to a broad range of vascular pathogens, including *Ralstonia solanacearum* (Denancé et al., 2013).

Through RNAi and CRISPR/Cas9 strategy, Koseoglou et al. inactivated the orthologous gene *SlWAT1* in tomato and demonstrated that this inactivation both reduced free auxin contents and ethylene synthesis in tomato stems and suppressed the expression of specific bacterial virulence factors of the vascular bacterium *Clavibacter michiganensis*.

In nature and in general, *Xylella fastidiosa* attacks and provokes severe damage on woody hosts for example on olive trees. To facilitate the study of the interaction of this bacterium with the plants, several herbaceous models have been tried. Barò et. al. demonstrated that *Nicotiana benthamiana* is a suitable model as the bacterium reaches a high population level after the inoculation and induce a rapid symptom development with low variability in the response. The same Authors demonstrated that *N. benthamiana* treated with the antimicrobial peptide BP134 or plants in which this peptide gene was transiently expressed showed reduction in disease severity provoked by *X. fastidiosa*. Antimicrobial peptides against plant pathogenic bacteria, *X. fastidiosa* included, are therefore possible candidates to develop novel biopesticides (Badosa et al., 2022).

Efflux pumps form part of the resistance mechanism employed by bacteria to permit their survival in the hostile plant chemical environment (Pasqua et al., 2019). Pun et al. studied in *Pectobacterium brasiliense* the effect of some efflux pump inhibitors and plant-derived phytochemicals on bacterial activity and found that PabN and NMP were the best efflux pump inhibitors. They suggested that the efflux pump system AcrAB-TolC plays an important role in survival and fitness of *P. brasiliense* in the plant environment and that its inhibition is a viable strategy for controlling bacterial pathogenicity.

Effective management of plant bacterial diseases is very difficult (Sundin et al., 2016; Sharma et al., 2022). Determining the identity of bacterial plant pathogens is essential to disease management, and this process is linked to bacterial taxonomy (Bull and Koike, 2015).

Gašic´et al. proposed a novel species *Brenneria izbisi* as causal agent of deep bark canker of young walnut trees. Two other *Brenneria* spp. are described on walnut: *B. nigrifluens* and *B. rubrifaciens* as agents of shallow-bark canker and deep bark canker, respectively. They represent a serious threat to walnut production by the weakening of trees and consequent reduction in the number of nuts and in timber production. A rapid and specific conventional PCR protocol to identify the novel species was also proposed.

Kałuz˙na et al. developed molecular tools for identification of *Xanthomonas arboricola* pv. corylina that could be used with PCR, qPCR, and Loop-mediated isothermal amplification. The authors validated each of the tools using genomic DNA isolated from pure cultures of the bacterium and DNA isolated from artificially inoculated and field-infected plant material. These fast and accurate identification and detection methods will aid in the diagnosis and management of bacterial blight of hazelnut in nursery stock tissues, nurseries, and in both young and established orchards.

Finally, Hugouvieux-Cotte-Pattat et al. presented a taxonomical update of the *Dickeya* genus, whose species attack a wide range of crops and ornamentals causing soft rot symptoms. They resumed knowledge on the genus *Dickeya* and added new data from phenotypic, genomic and phylogenetic analyses.

## Author contributions

RB: Writing – original draft, Writing – review & editing. CM: Writing – original draft, Writing – review & editing. VC: Writing – review & editing. MH: Writing – review & editing. GS: Writing – original draft. GS: Writing – review & editing.
